# An Audit of Accessibility and Actionability of Molecular Profiling for Patients with Cancer of Unknown Primary at a Tertiary Care Centre

**DOI:** 10.3390/clinpract16020039

**Published:** 2026-02-12

**Authors:** Khaled Abdulalem, Jonah Teich, Erika Martinez, Samuel D. Saibil

**Affiliations:** 1 Princess Margaret Cancer Centre, Toronto, ON M5G 2C1, Canada; khaled.abdulalem@uhn.ca (K.A.);; 2 Department of Medicine, University of Toronto, Toronto, ON M5S 1A1, Canada

**Keywords:** cancer of unknown primary, molecular analysis, molecular analysis, molecularly guided therapy, quality improvement

## Abstract

**Background/Objectives**: Cancer of unknown primary (CUP) remains a significant challenge in the field of oncology. Despite advances elsewhere in the field, there have been few advances in the treatment of CUP and correspondingly no improvements in patient survival. Recent studies utilizing molecular profiling, including next-generation sequencing (NGS), and molecularly targeted treatment of CUP have shown some promising initial results, but have yet to be integrated into the standard of care in most jurisdictions. This study aimed to assess the use of molecular characterization and targeted treatment of patients with CUP treated at Princess Margaret Cancer Centre (PMCC). **Methods**: This study is a retrospective audit of patients with CUP treated between January 2019 and April 2024 to build understanding of the accessibility and use of these molecular tools. **Results**: We found that 82% of the 28 patients identified received NGS analysis, though all received the results late in their disease course and all accessed molecular profiling via either clinical trials, a charitable access programme, or a privately source outside of the hospital network. Only 13% of the patients who received molecular analysis received any modification of care as a result of this profiling, and only as third line of treatment. **Conclusions**: Our data highlights a lag between current understanding and current practice, and identifies a possible area for improvement of patient care by standardizing the use of molecular analysis in the early workup and targeted therapy in the treatment of CUP.

## 1. Introduction

Cancer of unknown primary (CUP) is a metastatic disease in which no primary tumour can be identified following extensive diagnostic workup. CUP represents approximately 3–5% of all advanced cancer diagnoses, with a broad range of manifestations [1,2]. It can present as isolated single metastases or progress to widespread disease, involving multiple organs such as the liver, bones, lymph nodes, and lungs. The prognosis of CUP is generally poor, with a median overall survival of less than one year from diagnosis for those with high-risk (also called unfavourable) CUP [1]. Over the past decade, there have been few significant advances in the treatment of CUP, and there have correspondingly been no improvements in patient survival [2,3].

The standard treatment of CUP is empiric chemotherapy, usually based on platinum analogues and/or taxanes [4]. Some studies have focused on using tools such as histology, immunohistochemistry (IHC), and gene expression profiling to identify a likely tissue of origin. The hypothesized benefit of identifying the tissue of origin of CUP would be the ability to use site-specific therapy, as in treating the CUP as a cancer of the identified tissue of origin rather than the standard empiric therapy used for CUP. Although the expected benefits were well reasoned, the results of this work have so far been disappointing, with multiple meta-analyses finding no significant improvement in survival outcomes in patients with CUP treated with site-specific therapy compared to those treated with empiric chemotherapy [3,5].

A more recent innovation in cancer treatment is targeted therapy, in which treatments are guided by the presence of specific genetic mutations or overexpression of specific proteins, agnostic to the tissue of origin of the tumour. In many cases, these targeted therapies have been shown to result in better outcomes in cancer patients, with fewer and less severe adverse effects [6]. There has, as a result of the success of targeted therapies, been increased recent interest in exploring targeted therapy for many cancer types, including CUP. The focus of genetic research on CUP is twofold—finding mutations that are potentially targetable by novel medications, and identifying which of those mutations may already be targetable by currently existing medications [7,8,9,10,11,12].

Gatalica et al. studied 1806 CUP cases using next-generation sequencing (NGS) (47 genes), immunohistochemistry stain (23 markers) and in situ hybridization (7 genes). They found that 96% of CUP cases have mutations that are potentially targetable by specific therapies [8]. Similar results were found in another study that examined 3769 exons of 236 cancer-related genes and 47 introns of 19 genes commonly rearranged in cancer [9]. While most studies agree that a very large percentage of CUP cases have potentially targetable mutations, there is some disagreement on what percentage are currently actionable with existing, approved medications. Some studies have suggested that approximately 30% of CUP cases may have gene mutations targetable by current medications, while others have found that those numbers may be as high as 98% for the same criteria [10,11,12].

A recent and ongoing clinical trial, “Molecularly Guided Therapy Versus Chemotherapy After Disease Control in Unfavourable Cancer of Unknown Primary” (CUPISCO) [13], enrolled patients with unfavourable CUP and, in their test group, performed genomic analysis and provided molecularly guided therapy for those with mutations targetable by available medications who did not progress on three cycles of induction chemotherapy with a standard empiric regimen. In their patient population, approximately 27% of those who received gene testing had an actionable mutation. They further found that molecularly guided therapy in these patients improved progression-free survival versus standard empiric chemotherapy.

Despite promising preliminary data from the CUPISCO trial, CUP patients in Ontario do not have standardized access to NGS and molecular profiling as was performed on the CUPISCO trial. At the Princess Margaret Cancer Centre (PMCC), patients can access tumour mutational analysis and genetic profiling through various clinical trials and studies, though still not as a standardized, government-funded step in care. The percentage of CUP patients at PMCC that obtain advanced genetic profiling and the percentage of cases in which this data is used to inform and modify their treatment are not known. Accordingly, the goal of this project was to audit the charts of CUP patients treated at PMCC. We aimed to collect data on the extent to which cancer molecular profiling was able to be accessed at our centre, the mechanism through which it is accessed, and to what extent the results of gene profiling were used to inform and modify treatment. These data will then serve as a starting point towards building a standardized framework for accessing gene profiling for CUP patients and using genetic information to guide future treatment.

## 2. Materials and Methods

In this single-centre, retrospective, quality improvement project, data was collected from 28 patients who were diagnosed with CUP at PMCC’s CUP clinic between January 2019 and April 2024. Data was obtained from the patients’ electronic medical records, analyzed, and tabulated in a Microsoft Excel datasheet. The key data examined was biopsy and histology results, including tumour morphology and grade, as well as molecular analysis, including the means of access for each patient, which gene mutations were identified, and which patients received alternative treatments informed by their cancer’s genetic characteristics. Identified gene mutations from the study cohort were tabulated, along with the specific variants, the tier of the mutation, and how prevalent each mutation was in our sample group. Mutation tier is an indicator of how likely a gene mutation is to be linked to cancer, with tier 1 mutations having strong clinical significance, tier 2 mutations having potential clinical significance, tier 3 mutations having uncertain clinical significance, and tier 4 mutations being benign or likely benign. Tier 1, 2, and 3 mutations were recorded in this work.

Due to the low sample size, formal statistical analysis could not be performed at this stage of the study. Informal descriptive statistics were instead used to develop an early-stage understanding of patient and disease characteristics, diagnostic methods, clinicopathological features, and treatments.

## 3. Results

A total of 28 patients with unfavourable-risk CUP were identified by chart review and included in the analysis. The demographics of these patients are depicted in Table 1. The median age of patients was 63 years (total range from 22 to 84). Two patients (7%) were between the ages of 20 and 39 years, 10 patients (36%) were between the ages of 40 and 59, 15 patients (54%) were between the ages of 60 and 79, and one patient (4%) was between the ages of 80 and 100. In this sample of patients, the majority were female, with 22 female patients comprising 79% of the patients included in this study. Most patients also presented with extensive metastatic disease. In cases in which patients presented with multiple sites of metastases, biopsies were taken from the most accessible site. Liver (seven patients, 25%), lymph nodes (five patients, 18%), and omentum (four patients, 14%) were the most commonly biopsied sites. Brain, soft tissue, ovaries, skin, bone, and gallbladder were all less commonly biopsied, with a total of three each of brain and soft tissue biopsies, two each from ovaries and skin, and one each from gallbladder and bone. All patients included in this study had completed biopsies with comprehensive IHC analysis.

**Figure 1:**
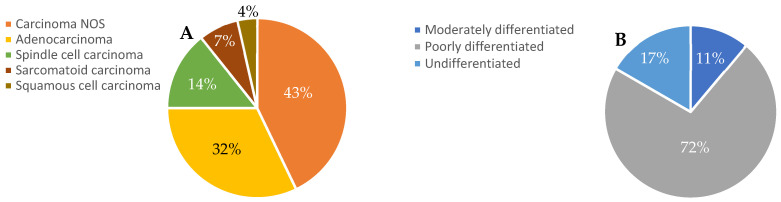
Summary of pathological assessment. The percentage of patient tumours with different pathological types (**A**) and tumour pathological grades (**B**) are depicted. *N* = 23 patients for each panel.

Upon this IHC pathological assessment, the plurality of tissue biopsy samples was consistent with carcinoma NOS (not otherwise specified) (12 patients, 43%). The next most common histological types were adenocarcinoma (nine patients, 32%), spindle cell carcinoma (four patients, 14%), sarcomatoid carcinoma (two patients, 7%), and squamous cell carcinoma (one patient, 4%). These results are depicted in Figure 1A. Tumour grade was also evaluated. The majority were poorly differentiated (13 patients, 72% of those with differentiation data, 46% overall), with the remainder being undifferentiated (three patients, 17% of those with differentiation data, 10% overall) and moderately differentiated (two patients, 11% of those with differentiation data, 7% overall). Data regarding differentiation was not available for the remaining 11 patients (representing 39% of the study group), and none of the available biopsy data showed well-differentiated tissue (Figure 1B). IHC has an important role in working up suspected CUP, since it can identify markers associated with certain tissue types and therefore aid in identifying a primary tumour site. In this study, 14 (50%) of the patient biopsies examined with IHC tested positive for cytokeratin 7 (CK7), distributed between adenocarcinoma in six patients and nonspecific carcinoma in five patients. A further eight (29%) of the patient biopsies tested positive for cytokeratin 19 (CK19), with an even distribution of four having histology consistent with adenocarcinoma and four with histology consistent with nonspecific carcinoma. Collectively, all of these data were consistent with the presentation of unfavourable-risk CUP and would warrant treatment with empiric chemotherapy in the first line.

**Figure 2:**
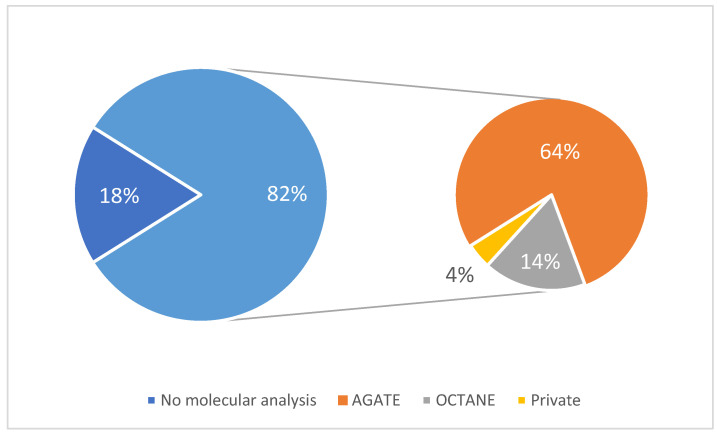
Prevalence of molecular analysis with distribution of access. The percentage of patients in the total study cohort (*n* = 28) that underwent NGS analysis (82%, light blue) is depicted on the left. The chart on the right depicts the means of access to obtain the NGS testing for those that were able to access it (*n* = 23).

Of the 28 patients, 23 (82%) underwent molecular analysis. At PMCC, molecular analysis is not yet fully integrated into the standard of care for CUP patients as it is not funded by the provincial healthcare insurance. Thus, access to NGS must be obtained through a variety of clinical trials and initiatives. Of the patients whose records were examined for this study, the majority (18 patients, comprising 64% of the total patients, and 78% of the patients who obtained molecular analysis) obtained their access to molecular analysis through the AGATE (Access to Genetic Advanced Testing) initiative, which was an institution-funded NGS initiative for patients with advanced solid tumour cancers. Another four patients (14% of the sample group, 17% of the patients who obtained molecular analysis) obtained access to testing through the OCTANE (Ontario-wide Cancer Targeted Nucleic Acid Evaluation) clinical trial, and one patient (4% of the sample group, 4% of the patients who obtained molecular analysis) purchased their own gene testing kit outside of the PMCC system, as depicted in Figure 2.

The majority of patients (21 of 23, 91%) who received molecular analysis had at least one identified genetic mutation. Of these patients, two (9% of those tested, 10% of those with identified mutations) possessed at least one identified tier 1 mutation, as defined as a variant with a strong clinical significance. An additional 17 patients (74% of those tested, 81% of those with identified mutations) possessed at least one tier 2 mutation, defined as a mutant with potential clinical significance, without any additional tier 1 mutations, and two patients (9% of those tested, 10% of those with identified gene mutations) possessed at least one tier 3 mutation, defined as a mutation without known clinical significance, without any tier 1 or tier 2 mutations. Despite this, only three patients of the 23 (13% of those who received molecular analysis) received modified treatment targeting one of their mutations (Table 2). One patient was identified as having the Tuberous sclerosis complex 2 (TSC2) mutated gene and was then treated with Everolimus as their third line of treatment. One patient had an epidermal growth factor receptor (EGFR) mutation and was then treated with lapatinib and capecitabine as their third line of treatment, and one patient had an identified isocitrate dehydrogenase 1 (IDH1) mutation and received ivosidenibe as their third line of treatment. All of these patients had to access these therapies via paying out of pocket and not via a clinical trial.

Some cancer-related genes are targetable by specific medications, but only for certain sequence variants of these genes. There were several cases in this small patient group in which a patient had a mutation in a targetable gene but did not have one of the sequence variants that was eligible for any targeted medication. These included one patient with a BRCA1 mutation with the sequence variant c.2521C>T, one patient with a BRCA2 mutation with the sequence variant c.5965T>C, two patients with PIK3CA mutations present with the sequence variants c.2176G>A and c.3140A>G, and one patient with a BRAF mutation with the sequence variant c.1454T>G. Other mutations included six patients with TP53 mutations, three with APC mutations, and two with KRAS and PTEN mutations. Other less frequently identified gene mutations were also found in these patients. Each patient had a mean of two identified mutations. A full list of mutations and sequence variants present in our patient group is listed in Table 3.

## 4. Discussion

Cancer of unknown primary still presents a challenge in terms of pathological assessment. In this project, the pathological and IHC analysis revealed staining patterns that did not align with any specific cancer origin, emphasizing the significant complexities associated with diagnosing cancers of unknown primary. The IHC stains utilized in the study, which are commonly applied to determine tissue origin by analyzing characteristic protein expression, failed to produce a unique or definitive pattern that could conclusively point to a primary site. Instead, the results demonstrated variability in marker expression, reflecting the heterogeneous nature of CUP and the challenge it poses in pathological assessment. This lack of a distinct staining profile highlights the limitations of current IHC techniques in these cases.

Molecular analysis was performed to investigate potential mutations that might shed light on the origin or behaviour of the cancer, as well as to guide treatment. The analysis revealed numerous gene mutations, reflecting the genetic complexity and heterogeneity of the disease. Despite the abundance of detected mutations, only three mutations, representing 13% of those who received this testing, were identified as being targetable with available medications. While the reliability of this number is limited by the sample size of this study, numbers cited by much larger studies [13] have shown a targetable mutation rate of approximately 30% of those tested, though this rate is still uncertain with a wide range available in publications [10,11,12,13]. This finding underscores the challenge of translating genomic data into effective treatment options, as many mutations do not yet have corresponding therapies or may lack clinical significance. It also highlights the importance of precision medicine in oncology, where identifying actionable mutations can help guide targeted treatment strategies, including the development of new medications based on common gene mutations. The results emphasize the need for ongoing research to expand the repertoire of therapeutic targets and improve the utility of molecular analysis in managing cancers with complex mutation profiles.

Assessing the magnitude of benefit from the targeted therapy is challenging, particularly given that it was administered as a third-line treatment. At this stage of therapy, patients often have more advanced disease and may have developed resistance to previous treatments, which can influence the effectiveness of subsequent interventions [14]. Additionally, the clinical benefit of targeted therapies may be confounded by the cumulative effects of prior therapies, the progression of the disease, and the general decline in patient health often associated with late-line treatment settings. These factors make it difficult to isolate and evaluate the true impact of the targeted therapy in improving outcomes such as tumour response, progression-free survival, or overall survival. This highlights the need for genetic testing to be integrated into earlier stages of care to avoid these confounding effects and potentially improve outcomes if these targeted therapies are proven to be at least non-inferior to the current standard of care.

Recently, the CUPISCO trial assessed the use of molecularly guided therapy (MGT) following 3 months of standard chemotherapy for patients with disease stabilization and demonstrated promising results in terms of progression-free survival in the MGT group. Their results further highlight the need to further study this field, and to better integrate molecular analysis into the early stages of CUP diagnosis as a step that could potentially improve outcomes in a sizeable portion of the patient population.

Access to molecular analysis for patients in PMCC was not consistently available throughout the study. A total of 18 patients underwent molecular analysis through the AGATE Program, which is part of a charitable access program which has subsequently closed. Additionally, the OCTANE clinical trial facilitated molecular analysis for four patients. One patient obtained their molecular analysis privately, presumably after failing to secure access through any of the options available at PMCC. Another five patients did not receive any molecular analysis. While a large percentage (82%) of the patients described in this study did receive molecular analysis, they received it late in treatment and did not have a consistent, easy, and standardized pathway. As Canada’s largest cancer hospital, access to these resources at PMCC is likely much more available than at other centres in Canada that receive less funding and have fewer available clinical trials. This clearly highlights a gap in patient care in which current practice lags behind current understanding. It therefore demonstrates an opportunity for improvement.

Despite the as-yet early stages of this research and not entirely conclusive results to date, the data that is available so far suggests that expanding and standardizing the access to molecular analysis in patients with CUP, in addition to improving future data collection and analysis, could improve the outcomes of many patients with this disease. The next step of this quality improvement project will be integrating standardized genetic testing into the early stages of CUP workups at PMCC. This will require coordination with the hospital genetics laboratories, but is unlikely to strain existing resources or require the acquisition or construction of new resources due to the small number of CUP patients relative to the total number of patients that require these facilities. Depending on the results of this follow-up study, further steps would likely involve the rollout of a standardized framework at other locations, or the further centralization of CUP patients to PMCC and other hospitals that have extensive pre-existing genetic facilities.

It is important to note that due to the small sample size of this project, the distribution of demographics, disease sites, histology, and specific mutations are likely to be skewed compared to those in the larger population. It is, however, the opinion of the authors that the overall pattern of access and usage of molecular profiling as described in this paper is representative of an identifiable healthcare gap.

## 5. Conclusions

While early results of targeted therapy in the treatment of cancer of unknown primary are promising, the current standard of care does not present a reliable framework for either the study or integration of this treatment modality. At PMCC, this manifests as late access, inconsistent access, and low use rates of genetic testing and targeted therapies.

By standardizing the use of genetic testing as part of the early workup of CUP, improvements can be made in patient outcomes and patient comfort, in addition to boosting the ability to analyze what benefits may be yielded from this new modality of treatment.

## Figures and Tables

**Table 1:** Patient demographics.

Patients		N (%)
Age		
	20 to 39 years of age	2 (7%)
	40 to 59 years of age	10 (36%)
	60 to 79 years of age	15 (54%)
	80 to 100 years of age	1 (4%)
Sex		
	Female	22 (79%)
	Male	6 (21%)
Biopsy site		
	Liver	7 (25%)
	Lymph node	5 (18%)
	Omentum/peritoneum	4 (14%)
	Soft tissue	3 (11%)
	Brain	3 (11%)
	Ovary	2 (7%)
	Skin	2 (7%)
	Gallbladder	1 (4%)
	Bone	1 (4%)

**Table 2:** Targeted mutations and medications used for treatment.

Molecular Mutation	Target Therapy	Line of Treatment
TSC2	Everolimus	3rd line
EGFR	Lapatinib	3rd line
IDH1	Ivosidenib	3rd line

**Table 3:** Mutations found by molecular analysis in patient group.

Mutation	Tier	Variant	Number of Patients
STK11	TIER II	c.158dup	1
TP53	TIER II	c.742C>T c.488A>G c.1009C>T	6
CREBBP	TIER II	c.6713G>C	1
SMARCB1	TIER II	c.781C>T	1
TSC2	TIER II	c.157G>A	1
CDKN2B	TIER II	-	1
POLD1	TIER II	-	1
MET:CAV1	TIER II	-	1
ERBB2	TIER II		1
NCOR1	TIER II		1
NF2	TIER II		1
ATACGATGGC	TIER II		1
PBRM1	TIER II	c.2335C>T	1
EGFR	TIER II	-	1
KIT	TIER II	-	1
PDGFRA	TIER II	-	1
IDH1	TIER I	c.394C>T	1
BCORL1	TIER II	c.1330delA	1
FGFR2	TIER I	-	2
TACC1	TIER II	-	1
CTNNB1	TIER II	c.97T>C	1
KRAS	TIER II	c.35G>A	2
PIK3CA	TIER II	c.2176G>A c.3140A>G	2
PTEN	TIER II		1
APC	TIER II	c.2532C>G c.4514delG c.8107C>T	3
BRCA1	TIER II	c.2521C>T	1
CCNE1	TIER II	-	1
POLE	TIER II	c.4513C>T	2
RAD51B	TIER II	-	1
BAP1	TIER II	c.567C>A	1
BRIP1	TIER II	c.3103C>T	1
NRAS	TIER II	c.182A>G	1
NF1	TIER II	c.2998C>T	1
BRAD1	TIER II	c.109A>G	1
CDK12	TIER III	c.2485G>A	2
		c.1747A>G	
ERBB2	TIER II	c.346G>A	1
HRAS	TIER II	c.59C>T	1
BRAF	TIER II	c.1454T>G	1
BRCA 2	TIER II	c.5965T>C	1

## Data Availability

The original contributions presented in this study are included in the article. Further inquiries can be directed to the corresponding author.

## Abbreviations

The following abbreviations are used in this manuscript:

CUP	Cancer of unknown primary
PMCC	Princess Margaret Cancer Centre
IHC	Immunohistochemistry
